# Effects of Foliar Calcium and Magnesium Application on Peach (*Prunus persica* L.) Nutrition Status and Fruit Quality Formation

**DOI:** 10.3390/plants15182873

**Published:** 2026-09-19

**Authors:** Chang Lu, Xudong Wang, Xiaoying Guo, Bilal Hussain, Shane Wang, Xiaoe Yang

**Affiliations:** 1 State Key Laboratory of Soil Pollution Control and Safety, Zhejiang University, Hangzhou 310058, China; 2 Key Laboratory of Environmental Remediation and Ecosystem Health, Ministry of Education (MOE), College of Environmental and Resources Sciences, Zhejiang University, Hangzhou 310058, China

**Keywords:** foliar calcium, foliar magnesium, chlorophyll, fruit quality, antioxidant activity, peach

## Abstract

Peach fruit quality—including sweetness, firmness, and nutritional value—is strongly determined by mineral nutrition, yet limited soil availability of calcium (Ca) and magnesium (Mg) under production activities often constrains high-quality fruit formation in juicy peach production. This study aimed to evaluate the effects of foliar application of Ca and Mg on leaf nutrient status and fruit quality in peach (*Prunus persica* L. Batsch). Seven treatments were established, including 0.1% (T1), 0.2% (T2), and 0.3% (T3) CaCl_2_ solutions, 0.1% (T4), 0.2% (T5), and 0.3% (T6) MgSO_4_ solutions, and a water-sprayed control (CK). All treatments were applied during the fruit development stage, and RNA sequencing analysis was performed on the highest-concentration MgSO_4_ treatment group. The results of this study showed that high-concentration treatments (0.3% CaCl_2_, T3; 0.3% MgSO_4_, T6) significantly increased leaf chlorophyll and Ca/Mg accumulation relative to CK. For Ca treatments, T3 significantly increased sucrose content, whereas T2 enhanced glucose accumulation and total phenolic content, indicating that appropriate CaCl_2_ supplementation may improve fruit quality through regulation of carbohydrate metabolism and antioxidant responses. T6 produced the greatest gains in soluble sugars, soluble solids, vitamin C, and antioxidant capacity, indicating a close link between leaf Mg status and carbohydrate accumulation. Transcriptomic analysis of peach leaves further revealed that Mg supplementation modulated genes involved in starch and sucrose metabolism, contributing to sugar accumulation. This study revealed that foliar application of 0.3% MgSO_4_ offers an effective strategy for improving peach fruit quality.

## 1. Introduction

Peach (*Prunus persica* L. Batsch) is one of the most economically important fruits in subtropical and warm temperate climates worldwide and is widely valued by consumers for its attractive flavor, juicy texture, and nutritional properties [1]. In China, honey peach cultivars, including Fenghua Yulu, are highly favored in fresh-fruit markets because of their distinctive aroma, high sweetness, and soft flesh texture. With increasing consumer demand for premium fruit, peach production has gradually shifted from yield-oriented cultivation toward the improvement of fruit quality and nutritional value [2]. Therefore, enhancing fruit quality has become a central objective in modern peach cultivation, as quality traits directly influence consumer acceptance, market competitiveness, and economic returns [3].

Under field production conditions, plant growth is often restricted owing to insufficient soil nutrient availability and poor plant nutrient uptake efficiency [4,5]. In production activities, continuous cultivation and high productivity requirements can result in substantial removal of available mineral nutrients from the soil. Long-term cultivation may further lead to nutrient depletion, nutrient imbalance, and reduced availability of essential elements, thereby limiting plant growth and fruit quality development [6]. Among them, Ca and Mg play distinct but complementary roles in fruit development and quality formation. However, Ca has limited mobility within plants and is predominantly transported through the xylem with the transpiration stream, resulting in poor redistribution from older tissues to developing organs. Therefore, Ca deficiency in rapidly growing tissues and fruits may occur despite sufficient calcium availability in other plant parts, highlighting the importance of effective calcium supplementation during fruit development. Calcium contributes to cell wall stability, membrane integrity, stress response, and fruit firmness [7]. Insufficient Ca supply is often associated with physiological disorders, fruit softening, cracking, and reduced commercial quality [8,9]. Mg, by contrast, is the central atom of chlorophyll and is essential for photosynthesis, carbon assimilation, carbohydrate metabolism, and assimilate transport [10,11,12]. Mg deficiency can reduce photosynthetic capacity and accelerate leaf senescence [13]. Therefore, inadequate Ca and Mg nutrition may restrict both the structural stability and metabolic basis of peach fruit quality.

Traditionally, mineral nutrient deficiencies in fruit trees have often been corrected through soil or rhizosphere fertilization strategies, including approaches designed to improve micronutrient acquisition in citrus roots [14]. Although soil application of Ca- and Mg-containing fertilizers can improve nutrient supply, its effectiveness is frequently restricted by soil-related factors. Nutrients applied to the soil may undergo fixation, precipitation, leaching, or immobilization, reducing their availability for plant uptake [15]. Moreover, root nutrient acquisition is strongly influenced by soil pH, moisture status, root distribution, and root physiological activity. Consequently, high fertilizer input does not always result in efficient nutrient absorption, which may reduce fertilizer use efficiency and increase environmental risks [16]. Foliar fertilization has emerged as a practical strategy for improving nutrient utilization efficiency and correcting mineral deficiencies during critical growth stages. Compared with rhizosphere soil fertilization, foliar fertilization enables nutrients to be supplied directly to aboveground tissues. Foliar fertilization improves nutrient use efficiency, yet its effectiveness is affected by formula composition, adjuvants, application timing, and weather conditions [17]. Foliar-applied nutrients can enter leaves through the cuticle, stomata, and other aqueous pathways, and their subsequent mobility depends on nutrient form and plant physiology [18]. Foliar fertilizers are widely used to alleviate nutrient deficiencies in crops during critical growth stages [19] and for biofortification to address micronutrient deficiency issues [20]. Studies have shown that foliar fertilization can increase the concentration of health-beneficial phytochemicals in fruits [21], as well as enhance fruit quality, nutrient composition, and antioxidant activity [22].

Nevertheless, the effectiveness of foliar fertilization depends strongly on application concentration, crop species, developmental stage, and field conditions. In commercial production, excessively low concentrations of a single mineral element may fail to alleviate nutrient deficiency, whereas overly high concentrations may reduce fertilizer utilization efficiency or increase the risk of foliar phytotoxicity. In addition, Ca and Mg regulate fruit quality through different physiological pathways: Ca mainly maintains tissue structural integrity and enhances stress tolerance, whereas Mg primarily promotes chlorophyll biosynthesis, photosynthetic carbon assimilation, and carbohydrate metabolism. Therefore, evaluating Ca and Mg as two independent foliar fertilization regimes under the same field management conditions is necessary to clarify their specific effects on leaf nutrient status, chlorophyll accumulation, and fruit quality attributes. The objective of this study was to investigate the effects of different concentrations of foliar-applied CaCl_2_ and MgSO_4_ on Fenghua Yulu peach under field conditions. Specifically, three concentrations of CaCl_2_ and three concentrations of MgSO_4_ were compared with a water-sprayed CK to evaluate changes in leaf mineral nutrient content, chlorophyll content, fruit external quality, sugar-acid flavor traits, vitamin C content, antioxidant capacity, and related metabolic responses. This study aims to provide a theoretical and practical basis for the scientific and efficient use of foliar Ca and Mg fertilization in Fenghua Yulu peach production.

## 2. Results

### 2.1. Mineral Nutrients and Sucrose Content in the Leaves

**Figure 1:**
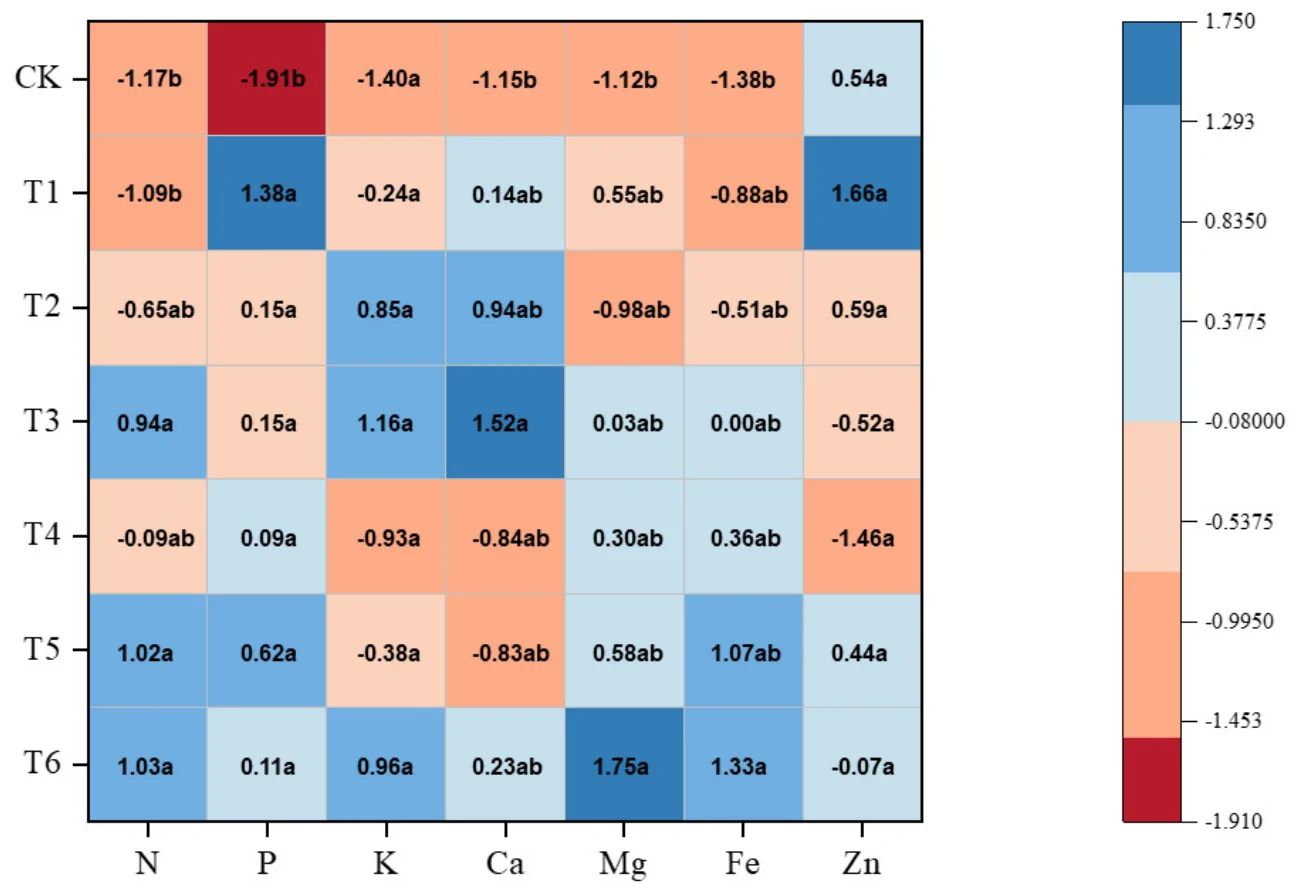
Effects of different foliar fertilizer treatments on leaf mineral nutrient contents of peach (*Prunus persica* L. cv. Fenghua Yulu). CK, water-sprayed control; T1, 0.1% calcium chloride; T2, 0.2% calcium chloride; T3, 0.3% calcium chloride; T4, 0.1% magnesium sulfate; T5, 0.2% magnesium sulfate; T6, 0.3% magnesium sulfate. The data were normalized before visualization. Values represent normalized relative levels of leaf mineral elements. Different letters indicate significant differences among treatments according to Duncan’s multiple range test at *p* < 0.05.

For foliar fertilizer treatments, compared with CK, as shown in Figure 1, all treatments (T1–T6) altered the contents of various mineral elements in peach leaves, but the influence patterns varied. All treatments increased leaf nitrogen content, with T3, T5, and T6 showing significant improvements; among these, T6 exhibited the highest nitrogen content, which was significantly increased by 14.1% compared with CK. All treatments significantly increased the phosphorus (P) content in leaves (*p* < 0.05). Among them, the T1 exhibited the most prominent effect, with a significant increase of 41.2% compared to CK.

The treatments affected the contents of Ca and Mg in leaves to varying degrees. Specifically, T3 showed the best promoting effect on Ca content, which was significantly increased by 48.6% compared with CK (*p* < 0.05). T6 had the most significant promoting effect on Mg content, with a significant increase of 55.9% compared to CK (*p* < 0.05). Although Ca and Mg contents showed increasing trends under several treatments, significant differences were only observed between specific treatments and CK. All treatment groups increased the iron (Fe) content in the leaves, with T6 showing the greatest increase, where Fe content rose significantly by 53% (*p* < 0.05). In contrast, the leaf zinc (Zn) content remained stable under all treatments. The Zn contents in leaves of T2–T6 decreased.

**Figure 2:**
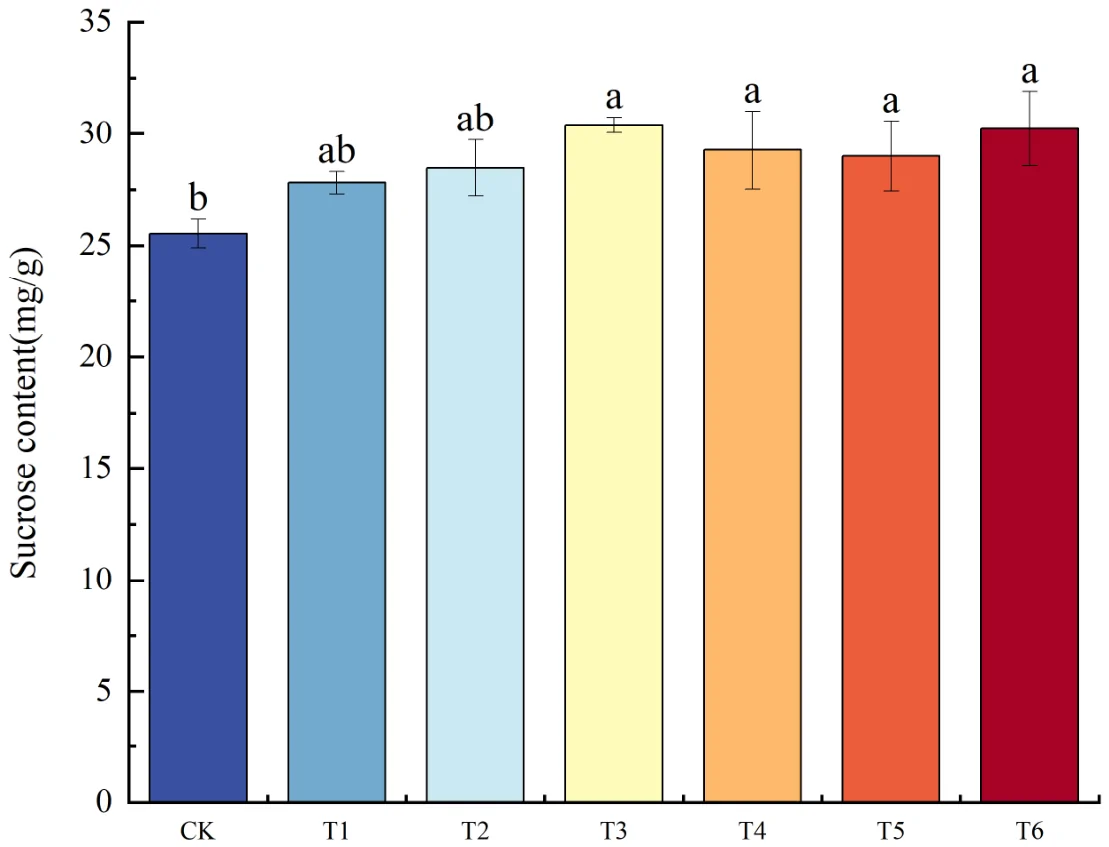
Effects of different fertilization treatments on sucrose content in leaves. Data are presented as means ± standard deviation SD (*n* = 3). Error bars represent SD. Statistical significance was determined using one-way ANOVA followed by Duncan’s multiple range test at *p* < 0.05. Different letters indicate significant differences among treatments.

Sucrose content in leaves was significantly higher in T3-T6 than in the CK, as shown in Figure 2. Among them, T3 exhibited the highest sucrose content, reaching 30.4 mg g^−1^, followed by T6 (30.23 mg g^−1^) and T4 (29.27 mg g^−1^). Compared with the CK, which had the lowest sucrose content, the sucrose contents in T3, T6, and T4 increased by 19.06%, 18.41%, and 13.62%, respectively (*p* < 0.05).

### 2.2. Chlorophyll Content in the Leaves

**Figure 3:**
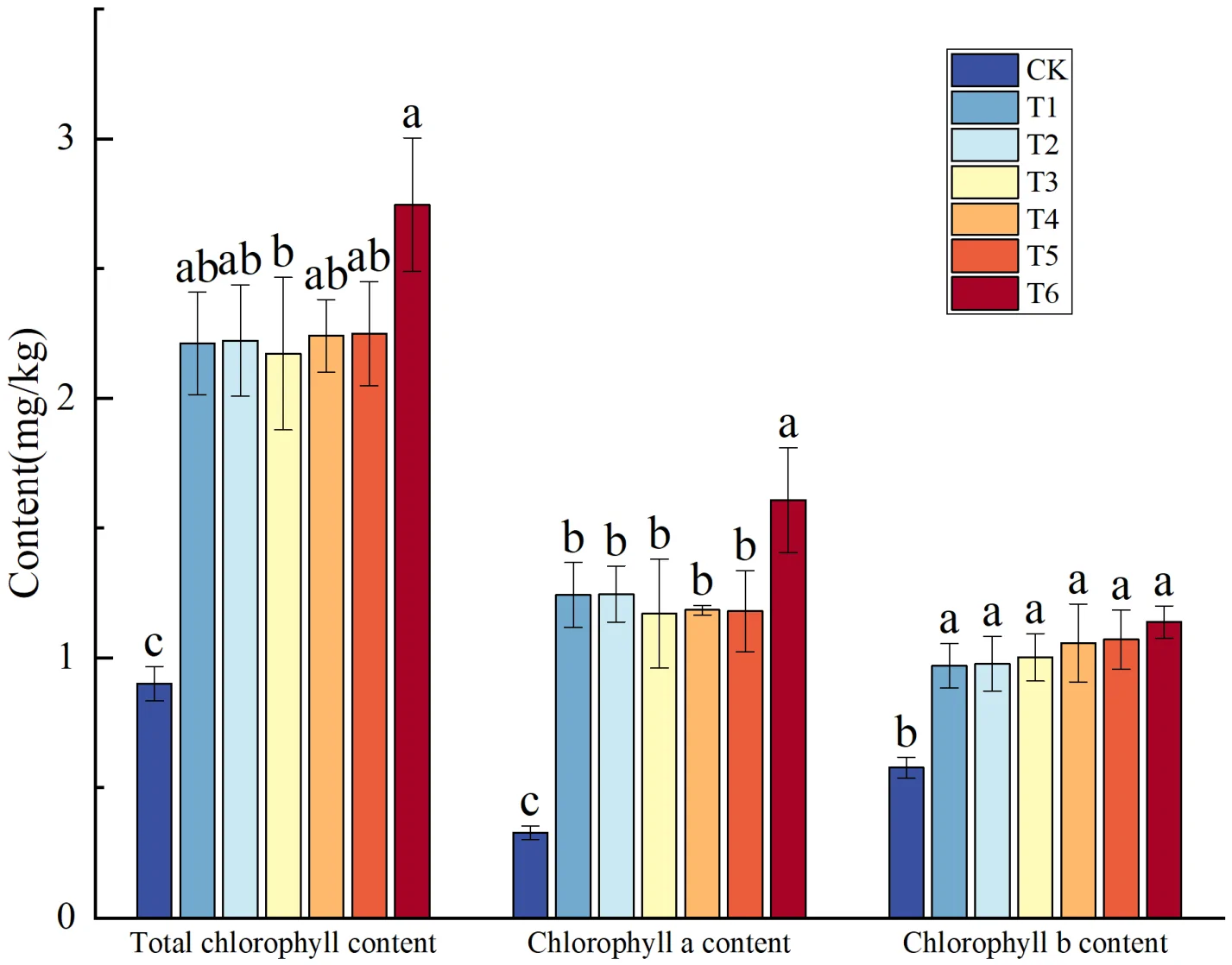
Effects of different fertilization treatments on contents of total chlorophyll, chlorophyll a, and chlorophyll b in leaves. Data are presented as means ± SD (*n* = 3). Error bars represent SD. Statistical significance was determined using one-way ANOVA followed by Duncan’s multiple range test at *p* < 0.05. Different letters indicate significant differences among treatments.

Foliar application of calcium and magnesium significantly affected the total chlorophyll content in peach leaves (Figure 3). Compared with CK, all fertilization treatments significantly increased the total chlorophyll content (*p* < 0.05). Among all treatments, T6 had the highest total chlorophyll content, which was significantly higher than that of the CK and other treatments. There were no significant differences between the T1–T5, but all were significantly higher than CK.

The change trend of chlorophyll a content was similar to that of total chlorophyll. The CK treatment had the lowest chlorophyll a content, and all fertilization treatments significantly increased the chlorophyll a content (*p* < 0.05). Among them, T6 had the highest chlorophyll a content, which was significantly higher than T1–T5, while there were no significant differences between the T1–T5 treatments. Foliar fertilization also significantly affected the chlorophyll b content in peach leaves. The chlorophyll b content of all calcium and magnesium treatment groups was significantly higher than that of the control. T6 had the highest chlorophyll b content, but there were no significant differences between the fertilization treatments.

### 2.3. Fruit Size and Shape

Table 1 shows the effect of different fertilization treatments on peach fruit weight. Compared with CK, the fruit weight of T6 was significantly increased by 9.0% (*p* < 0.05). There were no significant differences among T1–T6 treatments. Although fruit longitudinal length and equatorial diameter showed increasing trends under foliar fertilization treatments, no significant differences were observed among treatments. The fruit shape index, which reflects the ratio of fruit longitudinal diameter to transverse diameter, showed no significant differences among all treatments (Table 1). The fruit shape index of CK was 1.03, and the fruit shape indices of T1–T6 treatments were all close to 1, indicating that the fruit shape remained consistent under all treatments, with no significant differences (*p* > 0.05).

### 2.4. Fruit Sugar-Acid Flavor Quality

**Figure 4:**
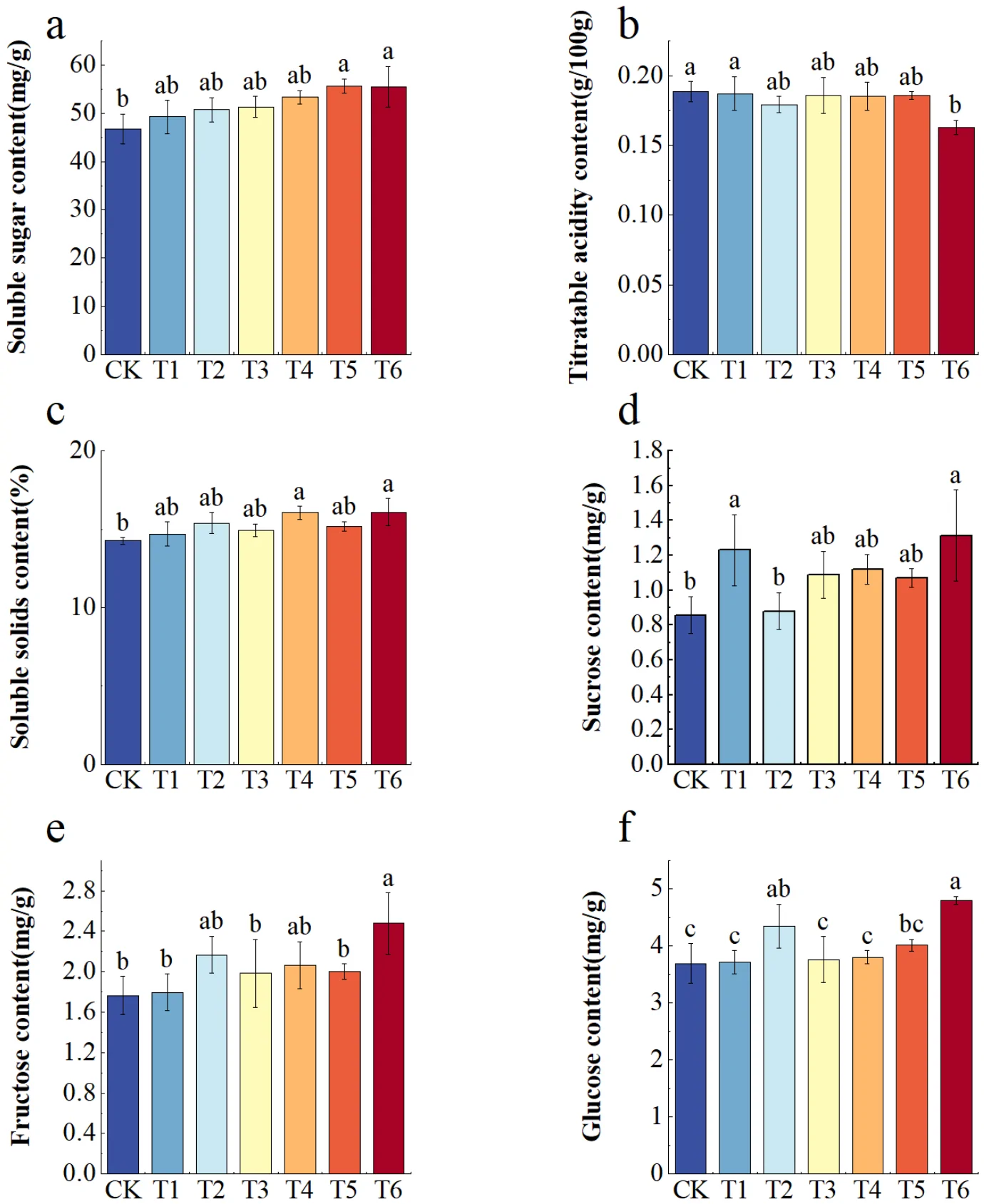
Effects of different fertilization treatments on fruit sugar-acid flavor quality indicators: soluble sugars (**a**), titratable acidity (**b**), total soluble solids (**c**), sucrose content (**d**), fructose content (**e**), glucose content (**f**). Data are presented as means ± SD (*n* = 3). Error bars represent SD. Statistical significance was determined using one-way ANOVA followed by Duncan’s multiple range test at *p* < 0.05. Different letters indicate significant differences among treatments.

Different fertilization treatments increased the soluble sugar content of Fenghua Yulu fruits (Figure 4a). Compared with CK, soluble sugar contents in T5 and T6 were significantly increased by approximately 19%. There are no significant differences in soluble sugar content among the T1, T2, T3, and T4.

The change trend of titratable acid content was also affected by fertilization treatments (Figure 4b). The titratable acid content of T6 was the lowest, which was significantly lower than that of CK and T1 (*p* < 0.05). Soluble solids content is a key indicator for evaluating sugar accumulation and flavor characteristics in fruits (Figure 4c). Across all fertilization treatments, the soluble solids content was significantly elevated in T6, peaking at 16.1%. As shown in Figure 4d–f, the glucose content of fruit ranged from 3.69 to 4.80 mg g^−1^, and significant differences were detected among different treatments (*p* < 0.05). T6 had the highest glucose content at 4.80 mg g^−1^, which was significantly higher than CK (3.69 mg g^−1^). Significant differences were also observed between CK and T2, while no significant difference existed between T2 and T6. CK presented the lowest glucose content and differed significantly from T2 and T6, suggesting that specific calcium and magnesium foliar fertilizer treatments may promote glucose accumulation in fruit. For fructose, T6 exhibited the maximum value of 2.48 mg g^−1^, which was markedly higher than CK, T1, T3, and T5. In general, magnesium foliar fertilizer increased fruit fructose content to varying degrees, and the content tended to rise with the increase in fertilizer concentration. In addition, sucrose content differed significantly across treatments (*p* < 0.05). T1 (1.23 mg g^−1^) and T6 (1.31 mg g^−1^) had the highest sucrose levels, which were significantly greater than CK (0.86 mg g^−1^). The sucrose contents of T3, T4 and T5 were 1.09, 1.12 and 1.07 mg g^−1^ respectively. Collectively, T1 and T6 showed significantly higher sucrose contents than CK, suggesting that appropriate foliar fertilizer treatments may promote sucrose accumulation in fruit.

### 2.5. The Vitamin C Content and Antioxidant Properties of the Fruit

**Figure 5:**
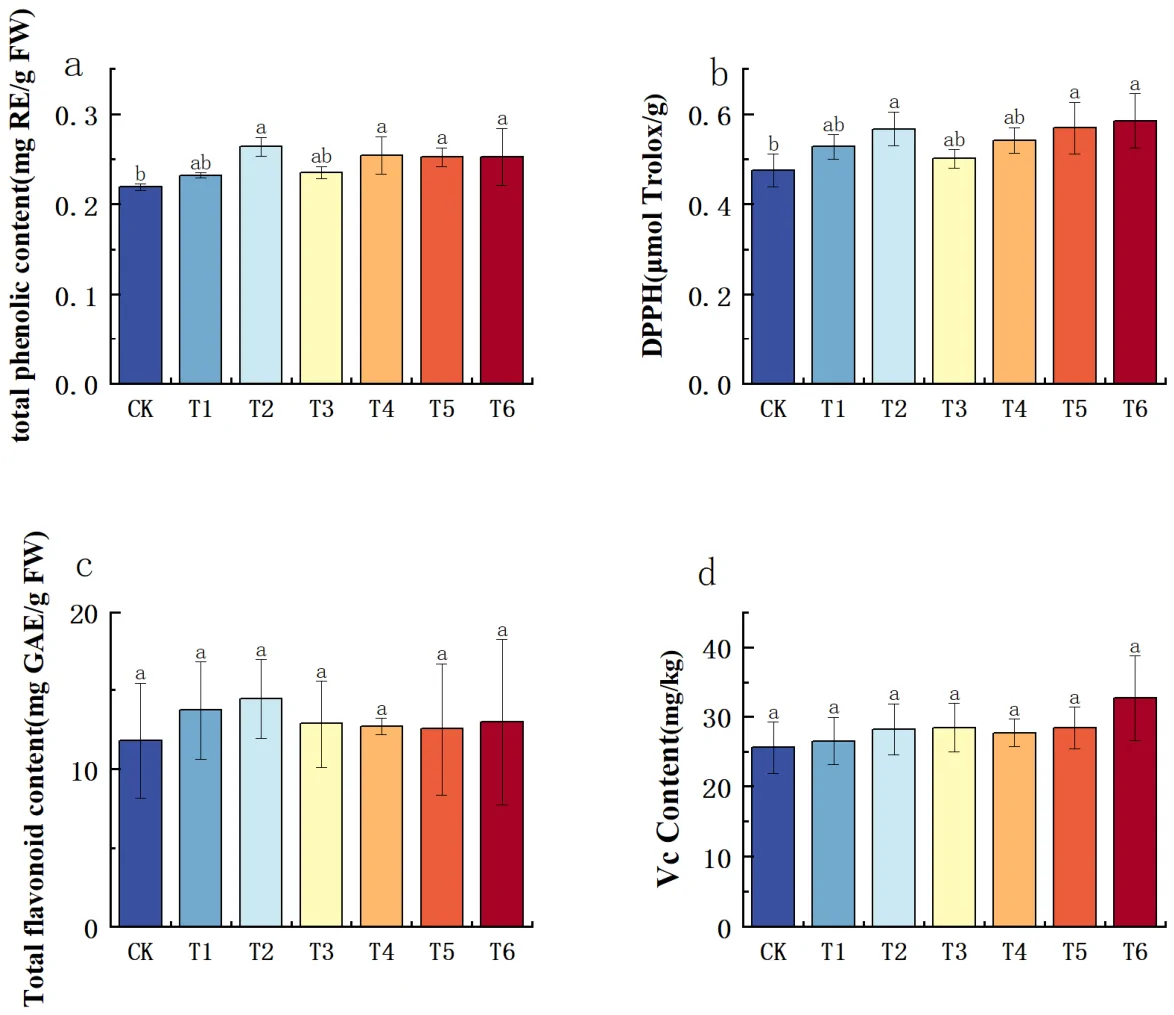
Effects of different fertilization treatments on fruit antioxidant properties and vitamin C content: total phenolic content (**a**), DPPH (**b**), total flavonoid content (**c**), and VC content (**d**). Data are presented as means ± SD (*n* = 3). Error bars represent SD. Statistical significance was determined using one-way ANOVA followed by Duncan’s multiple range test at *p* < 0.05. Different letters indicate significant differences among treatments.

The total phenolic content of peach fruits varied among different foliar fertilization treatments (Figure 5a). The total phenolic content in T2 fruits reached 0.26 mg gallic acid equivalents (GAE) g^−1^ FW, an increase of about 20% compared with the CK group, indicating that the application of calcium foliar fertilizer can effectively promote the accumulation of phenolic substances in the fruit. The total phenolic content of T2, T4, T5, and T6 ranged from 0.23 to 0.26 mg GAE g^−1^ FW, all significantly higher than CK (*p* < 0.05). The 2,2-diphenyl-1-picrylhydrazyl (DPPH) radical scavenging rate of the fruits reflects their antioxidant capacity (Figure 5b). The results showed that the DPPH radical scavenging rate of fruits in the T2, T5, and T6 foliar fertilizer treatments was higher than that of the CK group, with the T6 treatment recording the highest value. This indicates that foliar fertilizer treatment significantly enhanced the antioxidant capacity of the fruits. There were differences in total flavonoid content among fruits under different treatments (Figure 5c). The total flavonoid content of peach fruits showed variations among different foliar fertilization treatments. Treatment T2 showed the numerically highest total flavonoid content, reaching 14.47 mg rutin equivalents (RE) g^−1^ FW; however, no significant differences were detected among treatments. Effects of different foliar fertilization treatments on fruit vitamin C content are presented in Figure 5d. Vitamin C contents were numerically higher under the fertilization treatments than under CK, but the differences were not statistically significant. T6 showed the numerically highest vitamin C content, although no significant difference was detected among treatments.

### 2.6. The Content of Calcium and Magnesium in the Fruit

**Figure 6:**
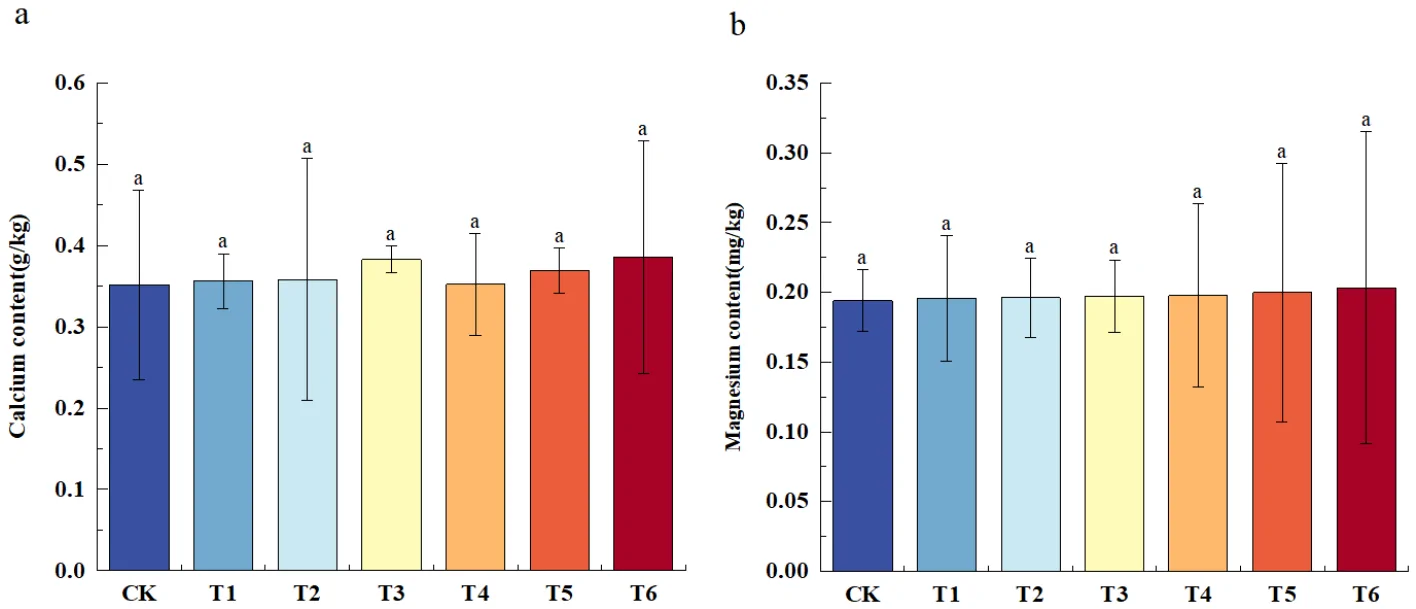
Effects of different fertilization treatments on fruit Ca (**a**) and Mg (**b**) contents. Data are presented as means ± SD (*n* = 3). Error bars represent SD. Statistical significance was determined using one-way ANOVA followed by Duncan’s multiple range test at *p* < 0.05. Different letters indicate significant differences among treatments.

The contents of Ca and Mg in Fenghua Yulu fruits were affected by different fertilization treatments (Figure 6). Numerically, T6 showed the highest Ca and Mg contents among the treatments; however, these differences were not statistically significant. Although foliar fertilization resulted in numerically higher fruit Ca contents under T1–T5 treatments compared with CK, the differences among treatments were not statistically significant. Similarly, fruit Mg contents showed increasing trends under foliar fertilization treatments, but no significant differences were detected among treatments.

### 2.7. Principal Component Analysis of Peach Fruit Quality Under Different Treatments

To explore the multivariate relationships among fruit quality traits, principal component analysis (PCA) was performed using the data from 21 independent experimental units and 10 fruit quality indicators. The individual replicate values, rather than treatment means, were used as observations in the PCA. Prior to PCA, the variables were standardized to eliminate differences in measurement scales. The KMO value of (0.637 > 0.6) and the significant Bartlett’s test of sphericity (*p* < 0.001) confirmed the suitability of the dataset for PCA. As shown in (Table 2).

Based on eigenvalue decomposition and variance partitioning, the cumulative variance explained by the first two principal components (PC1 and PC2) reached 63.5%, with PC1 and PC2 accounting for 45.5% and 18.0%, respectively. This indicates that the first two principal components effectively capture the majority of characteristics and quality information inherent in the original data. A loading matrix was subsequently employed to quantify the contribution of each quality indicator to the respective principal components. PC1 had an eigenvalue of 4.55, accounting for 45.5% of the total variance, and was primarily driven by soluble sugar content, single fruit weight, fruit transverse diameter, fruit longitudinal diameter, DPPH radical scavenging activity, and total phenolic content. PC2 had an eigenvalue of 1.8, contributing 18% of the total variance, and was primarily associated with titratable acidity, glucose, and fructose. PC3 had an eigenvalue of 1.3, explaining 13% of the variance, and was mainly associated with sucrose content.

**Figure 7:**
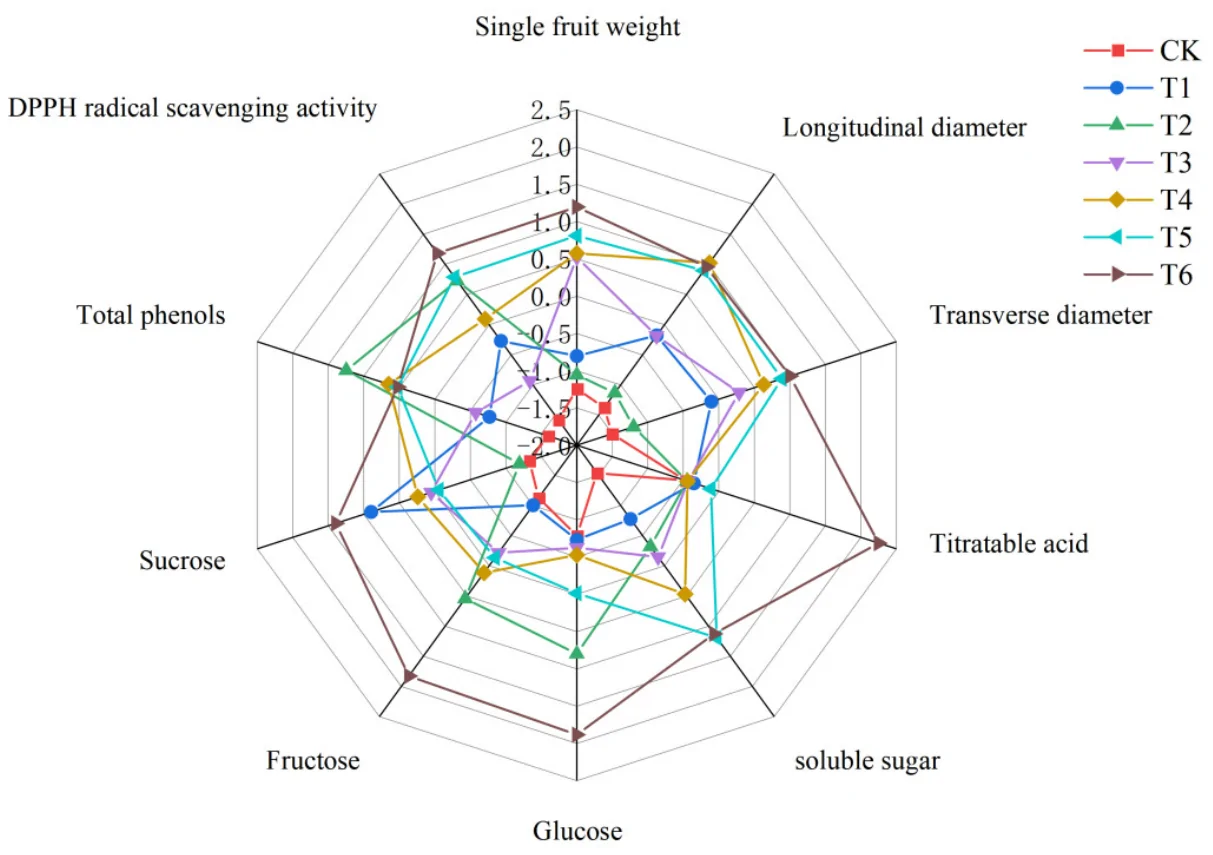
Radar chart evaluation of comprehensive fruit quality indicators under different foliar fertilizer treatments. All indices were standardized via Z-score transformation consistent with the PCA dataset. Titratable acidity was inversely transformed before plotting.

To visualize the differences in fruit quality characteristics among foliar-applied calcium and magnesium fertilizer treatments at different concentrations, a radar chart (Figure 7) was constructed based on ten standardized quality indicators. Compared with CK, foliar fertilization treatments showed distinct patterns of variation among fruit quality attributes. CK exhibited relatively lower standardized values for several quality indicators, whereas some fertilization treatments showed higher values for specific traits. Among all treatments, T6 displayed a relatively favorable overall performance, particularly in sugar-related indicators, suggesting its potential effectiveness in improving peach fruit quality under the conditions of this study.

### 2.8. Comprehensive Transcriptome Sequencing Analysis of Leaves Treated with 0.3% MgSO_4_

**Figure 8:**
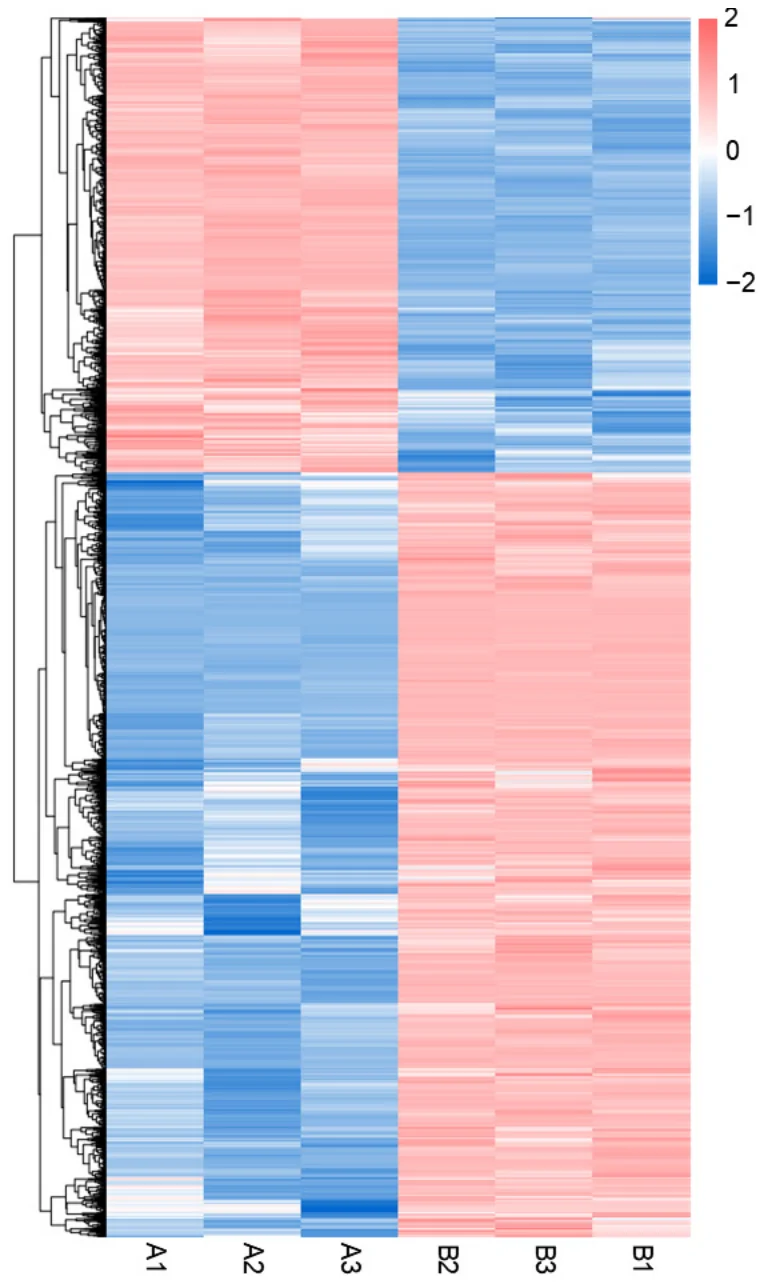

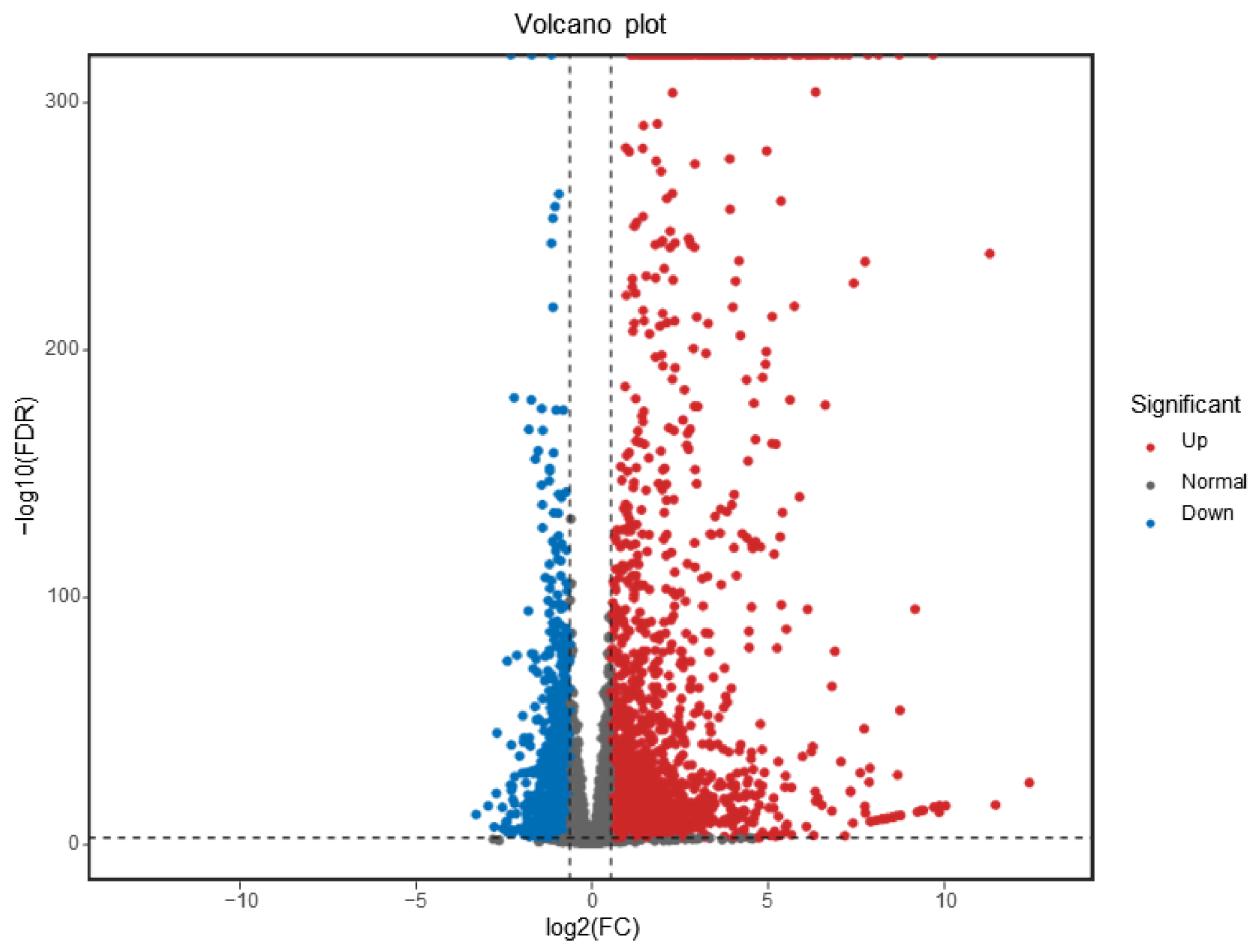
Transcriptomic analysis of peach leaves sprayed with 0.3% magnesium sulfate (A-CK, B-T6).

Leaves are the primary source organs for photosynthate production, and transcriptional changes in leaves may affect carbon assimilation and assimilate export to sink organs such as fruits. To explore molecular responses associated with the superior performance of T6, transcriptomic analysis was performed only for T6 and CK (Figure 8). Compared with CK, 0.3% MgSO_4_ treatment was associated with substantial differences in leaf gene expression profiles. A total of 3590 differentially expressed genes (DEGs) were identified, including 2250 upregulated genes and 1340 downregulated genes.

**Figure 9:**
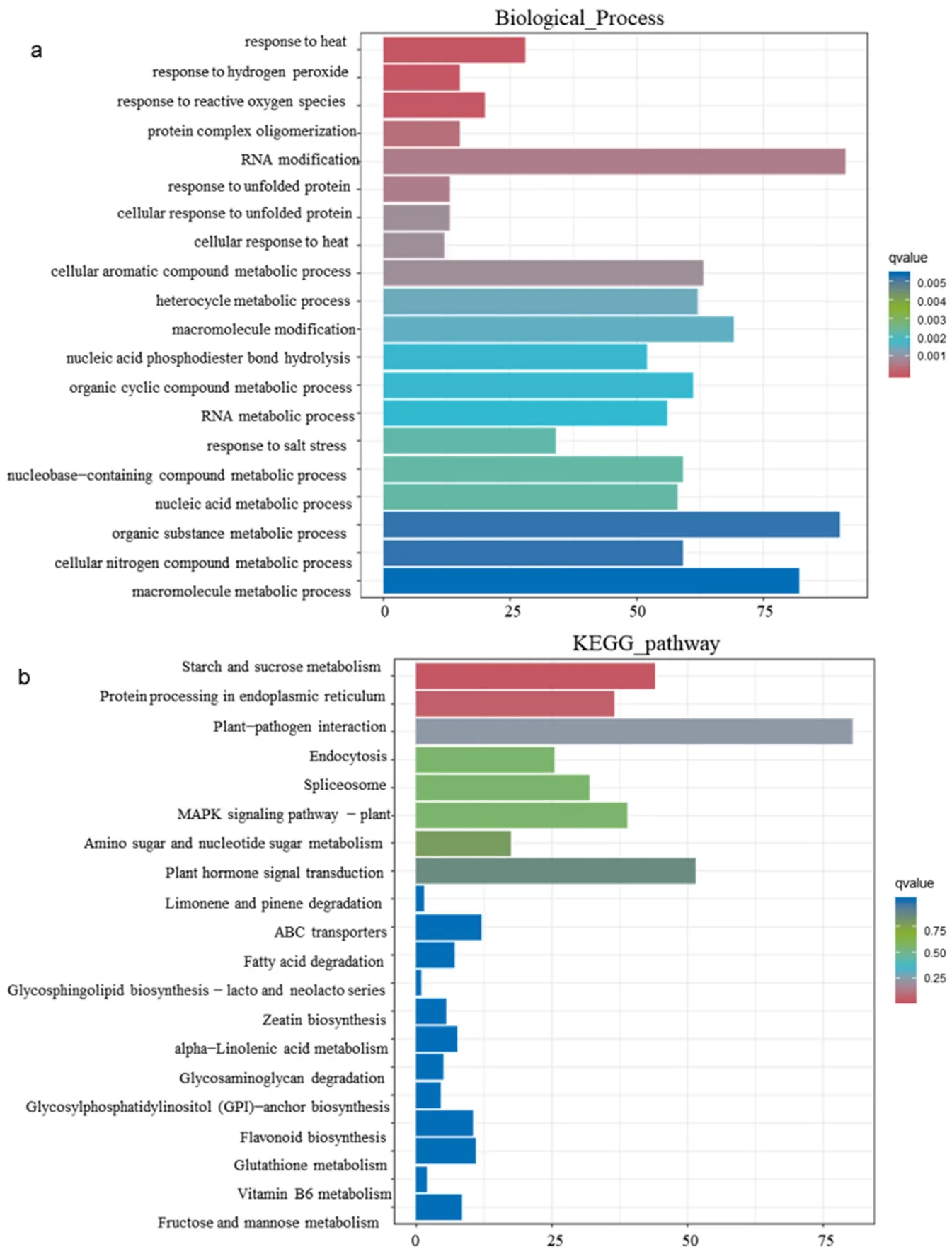
Top 20 enriched GO terms and KEGG pathways of differentially expressed genes in Fenghua Yulu leaves treated with 0.3% MgSO_4_ foliar spray. (**a**) The top 20 significantly enriched GO terms of differentially expressed genes under 0.3% MgSO_4_ treatment; (**b**) The top 20 significantly enriched KEGG pathways of differentially expressed genes under 0.3% MgSO_4_ treatment.

Furthermore, GO and KEGG enrichment analyses were conducted for the identified DEGs. GO enrichment analysis demonstrated that the DEGs induced by MgSO_4_ treatment were mainly associated with biological processes involved in cellular metabolism and environmental responses. In the Biological Process category (Figure 9a), DEGs were predominantly enriched in metabolic process, cellular process, response to stimulus, biological regulation, and macromolecule modification, suggesting that Mg application triggered extensive transcriptional reprogramming related to cellular activity and metabolic regulation. In addition, enrichment of terms associated with response to reactive oxygen species, response to oxidative stress, and protein processing indicated that magnesium treatment may also influence cellular redox homeostasis and stress adaptation.

KEGG enrichment analysis further revealed that Mg-responsive DEGs were mainly distributed among pathways involved in metabolism, genetic information processing, environmental information processing, and cellular processes (Figure 9b). Among the identified pathways, starch and sucrose metabolism was the pathway that remained statistically significant after multiple-testing correction, with an enrichment factor of 1.42 and 88 associated DEGs. Starch and sucrose metabolism was among the most prominently enriched pathways. Because this pathway is involved in carbohydrate synthesis, degradation, and conversion, the enrichment pattern suggests that MgSO_4_ treatment was associated with transcriptional changes in carbohydrate metabolism in peach leaves. Given that soluble sugars contribute to peach fruit sweetness and quality, these transcriptional changes may be related to the differences in fruit sugar accumulation observed in the present study.

### 2.9. Transcriptomic Analysis Reveals Magnesium-Induced Regulation of Carbohydrate and Secondary Metabolism Pathways in Peach Leaves

**Figure 10:**
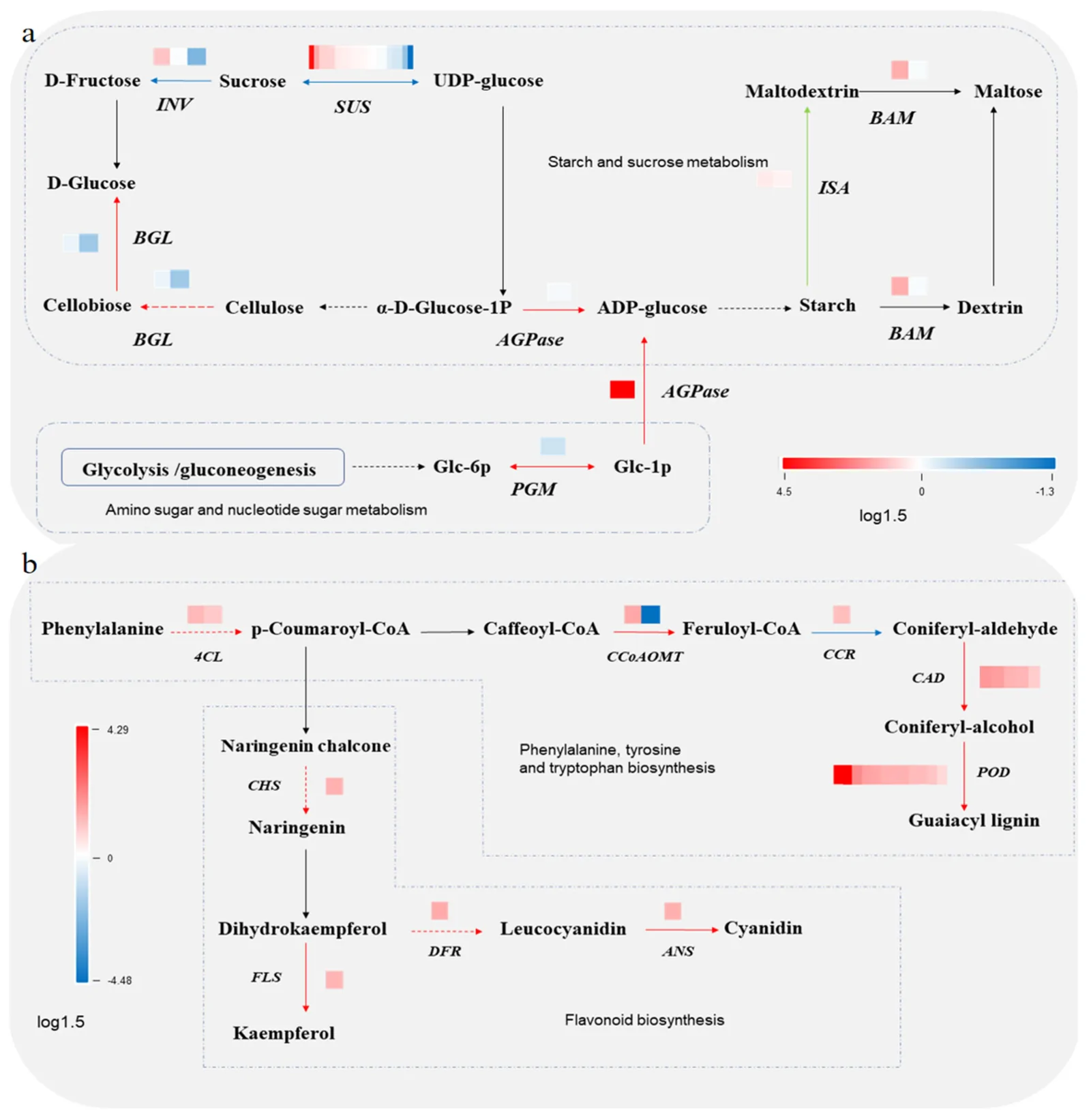
Overview of transcriptional changes in leaf carbohydrate and secondary metabolism under magnesium foliar fertilization. (**a**) Transcriptional profiles of genes involved in starch and sucrose metabolism, glycolysis/gluconeogenesis, and amino sugar and nucleotide sugar metabolism. (**b**) Transcriptional profiles of genes involved in phenylpropanoid, lignin, and flavonoid biosynthesis pathways. The regulatory pathways are constructed based on the KEGG database, with modifications. The multicolored horizontal bars (heatmaps) adjacent to the gene names represent the relative expression levels (log1.5-fold change) of individual transcripts or gene members in peach leaves under T6 compared to CK. Dashed arrows in the figure represent omitted intermediate reaction steps. Red arrows indicate upregulated genes, blue arrows correspond to genes with divergent expression (partial upregulation and partial downregulation), and green arrows denote downregulated genes. Abbreviations: INV, β-fructofuranosidase (invertase); SUS, sucrose synthase; BGL, β-glucosidase; AGPase, ADP-glucose pyrophosphorylase; PGM, phosphoglucomutase; BAM, β-amylase; ISA, isoamylase; 4CL, 4-coumarate-CoA ligase; CCoAOMT, caffeoyl-CoA O-methyltransferase; CCR, cinnamoyl-CoA reductase; CAD, cinnamyl alcohol dehydrogenase; POD, peroxidase; CHS, chalcone synthase; DFR, dihydroflavonol 4-reductase; FLS, flavonol synthase; ANS, anthocyanidin synthase.

Transcriptomic profiling was performed on peach leaf samples collected from magnesium foliar fertilizer treatments and clean water controls. Divergent expression patterns were observed for gene family members of INV and SUS under magnesium application, with partial transcripts upregulated and others downregulated. The enzyme INV (β-fructofuranosidase) catalyzes the irreversible hydrolysis of sucrose into glucose and fructose. SUS is a bidirectional enzyme that mediates both sucrose synthetic and degradative reactions (Figure 10a).

Higher transcript abundance of BGL under magnesium fertilization suggests a potential alteration in carbohydrate-related processes in leaves. The gene encoding ADP-glucose pyrophosphorylase (AGPase, the rate-limiting enzyme of starch biosynthesis) was markedly upregulated, alongside elevated expression of PGM.

Genes encoding isoamylase (ISA) and β-amylase (BAM) underwent contrasting transcriptional regulation: ISA expression was suppressed, whereas upregulated BAM catalyzes the breakdown of amylose and maltodextrin into maltose and dextrin. The gene encoding 4CL, an important enzyme in the phenylpropanoid pathway, was significantly upregulated. This change suggests a potential alteration in phenylpropanoid biosynthesis and the conversion of hydroxycinnamic acid derivatives into activated CoA esters. Meanwhile, upregulated CCoAOMT expression may be associated with changes in the biosynthesis of feruloyl-CoA and related phenylpropanoid compounds. These transcriptional responses may contribute to the regulation of phenylpropanoid metabolism; however, they do not by themselves demonstrate increased metabolic flux or substrate accumulation.

In the lignin biosynthetic branch, genes encoding CCR exhibited differential expression, while CAD transcripts were markedly upregulated. In addition, POD expression was significantly increased. The coordinated transcriptional responses of these genes suggest that magnesium application may influence lignin biosynthesis and cell wall-related processes (Figure 10b).

Furthermore, flavonols, including quercetin and kaempferol derivatives, are important antioxidant metabolites that contribute to fruit nutritional quality. The increased expression of key genes such as CHS and FLS suggests that magnesium application may regulate flavonol biosynthetic pathways. These transcriptional changes are consistent with the increased phenolic accumulation and antioxidant capacity observed in Mg-treated peach fruits, but further biochemical or enzymatic validation would be required to establish a direct relationship between gene expression and flavonol biosynthetic activity.

## 3. Discussion

The results of this study showed that foliar application of calcium and magnesium fertilizers at varying concentrations significantly increased the nitrogen, phosphorus, and potassium content in the leaves of Fenghua Yulu. Nitrogen promotes the synthesis and accumulation of sugars by enhancing photosynthetic capacity, whereas phosphorus and potassium are involved in the regulation of fructose content and sugar-acid ratio, which are key indicators of fruit flavor quality [23]. Consistent with the findings of this study, previous research has indicated that foliar application of Ca and Mg fertilizers can promote the accumulation of mineral elements such as Ca, Mg, and boron (B) in apples [24,25], further validating the positive effects of Ca and Mg foliar fertilization on mineral element accumulation in fruit crops. However, the effects of foliar nutrient application were strongly concentration-dependent, indicating that an appropriate nutrient level is required to maximize physiological benefits. Insufficient nutrient supply may fail to satisfy metabolic demands, whereas excessive application may disturb ionic balance and nutrient interactions, ultimately limiting plant performance [26].

Ca plays essential roles in plant growth and fruit development through both structural and signaling functions. As a structural nutrient, Ca contributes to cell wall stabilization by forming calcium–pectate complexes and maintaining membrane integrity, which is particularly important for cell expansion, fruit enlargement, and firmness maintenance. In addition, Ca^2+^ functions as a critical secondary messenger involved in regulating plant responses to environmental stimuli and coordinating various physiological processes [9]. Previous studies have shown that exogenous calcium application can help maintain leaf chlorophyll content and photosynthetic performance [27], consistent with the results of this study. The results of this study further indicated that foliar application of Ca fertilizers exerted a positive effect on improving external quality indicators of Fenghua Yulu, such as fruit weight and fruit diameter, which was in agreement with previous research findings [28]. In the present study, foliar calcium application increased fruit weight and soluble sugar content to varying degrees, while reducing titratable acidity, thereby improving the sugar–acid ratio. Foliar spraying of Ca before harvest enhanced the contents of vitamin C, total soluble solids (TSS), and phenolic compounds [29]. The beneficial effects of Ca were closely associated with application concentration. The appropriate Ca concentration likely provided sufficient Ca^2+^ for cell wall formation, membrane stabilization, and metabolic regulation, while avoiding potential antagonistic effects with other cations. Similar concentration-dependent responses have been reported in peach [30]. These findings suggest that the improvement of peach fruit quality by Ca application is not simply determined by increasing Ca availability but depends on achieving an appropriate balance between Ca supply and plant physiological requirements. It also indicates that appropriate concentrations of Ca foliar fertilization can more effectively promote carbohydrate synthesis and distribution, thereby optimizing fruit flavor quality.

Mg primarily regulates fruit quality through its fundamental role in photosynthetic carbon assimilation. Mg is the central atom of chlorophyll molecules and is also required for the activation of many enzymes involved in carbohydrate metabolism, including ribulose-1,5-bisphosphate carboxylase/oxygenase (Rubisco). Mg, as a central component of chlorophyll, participates in chlorophyll biosynthesis, thereby facilitating the occurrence of photosynthesis [31]. Under different magnesium concentration treatments, T6 exhibited the most significant effect with the highest chlorophyll content. This result indicates that an appropriate Mg concentration effectively enhanced chlorophyll synthesis and photosynthetic activity, thereby increasing the production of photoassimilates available for fruit development. The concentration-dependent response observed under Mg application may be attributed to the requirement of Mg for maintaining chloroplast function and metabolic homeostasis. At insufficient Mg levels, chlorophyll biosynthesis and enzyme activation are restricted, limiting photosynthetic carbon fixation. Conversely, excessive Mg supply may interfere with the uptake and utilization of other nutrients due to ionic competition. Therefore, moderate Mg supplementation can maximize photosynthetic efficiency and carbohydrate accumulation. Similar dose-dependent responses have been reported in fruit trees. In navel orange, approximately 150 g MgO/plant provided the optimal improvement in yield and sugar accumulation, while higher application rates showed limited additional benefits [32]. Likewise, foliar Mg application in grapevine showed an optimal concentration for enhancing photosynthetic performance, whereas higher Mg levels provided limited additional benefits [33]. It should be noted that Ca and Mg were supplied as CaCl_2_ and MgSO_4_, respectively. Therefore, the accompanying Cl^−^ and SO_4_^2−^ ions may also have contributed to the observed physiological responses. Cl^−^ participates in photosynthetic and osmotic regulation, while SO_4_^2−^ provides sulfur for the synthesis of sulfur-containing metabolites. Although their individual contributions were not separately evaluated, the observed responses may reflect the combined effects of the supplied salts and their respective nutrients.

Although both Ca and Mg treatments improved fruit quality, their regulatory mechanisms were fundamentally different. Ca mainly contributes to fruit development by strengthening cell wall structure, maintaining membrane stability, and activating Ca^2+^-dependent signaling pathways. In contrast, magnesium primarily promotes photosynthetic carbon fixation by regulating chlorophyll formation, enzyme activity, and carbohydrate metabolism. Therefore, the improvement of fruit quality caused by Ca and Mg foliar fertilization may result from complementary physiological regulation rather than identical mechanisms. Ca mainly affects fruit structural development and quality maintenance, whereas Mg enhances carbon assimilation and provides metabolic substrates for fruit growth.

Foliar application of magnesium fertilizer had a positive effect on improving fruit weight and diameter, as well as other appearance quality traits of ‘Fenghua Yulu’, which was consistent with previous studies [34]. Among all magnesium treatments, 0.3% MgSO_4_ showed the most significant effect on increasing fruit weight, and also significantly increased soluble sugar content while reducing titratable acidity, thereby improving the sugar–acid ratio. Regarding individual sugar components, significant differences were observed among treatments, with the T6 treatment showing the most pronounced effect. The enhanced sugar accumulation following Mg application may be attributed to improved photosynthetic carbon fixation and increased transport of assimilates from source leaves to developing fruits. Mg improved the accumulation of carbohydrates in fruits by regulating plant metabolic processes, thereby further increasing the nutritional and commercial value of fruits [35]. Relevant studies have shown that foliar application of Mg fertilizer significantly increased the TSS content of pomelo [36]. These findings are consistent with the results of this study. In addition to improving mineral nutrient accumulation and fruit physical quality, Ca and Mg foliar applications also positively influenced the antioxidant capacity of Fenghua Yulu. T2 and T6 treatments significantly increased the contents of total phenolics and DPPH radical scavenging activity, suggesting an enhancement of the fruit’s antioxidant defense system. This finding is consistent with previous reports [37,38]. This enhancement may be related to the roles of Ca and Mg in maintaining cellular stability and regulating metabolic pathways associated with antioxidant compound synthesis.

Metabolomic studies have also shown that Mg may play a key role in regulating the metabolic expression of phenolic compounds and flavonoids in various plant species. Studies on apple peel have demonstrated that appropriate Mg application in ‘Red Fuji’ apple trees significantly increased the expression of genes and transcription factors related to anthocyanin biosynthesis, suggesting a potential effect on the expression of phenolic compounds and flavonoids [39]. In this study, transcriptomic analysis of peach leaves was only conducted for the 0.3% MgSO_4_ treatment, since it showed the most significant improvement in leaf nutrient status, chlorophyll content, carbohydrate accumulation, and fruit quality. The transcriptomic results suggested that Mg treatment may be associated with changes in carbon assimilation and metabolic pathways, potentially contributing to altered carbohydrate metabolism and source–sink relationships. These transcriptional responses may be associated with the enhanced sugar accumulation, reduced acidity, and improved antioxidant capacity observed in Mg-treated peach fruits. However, transcript abundance alone does not directly demonstrate changes in metabolic flux, enzyme activity, or metabolite accumulation. The objective of transcriptomic analysis was to explore potential molecular responses associated with Mg-induced quality improvement rather than to comprehensively compare all nutrient treatments. We acknowledge that the absence of combined Ca and Mg treatments limits our understanding of potential interactions between these two nutrients. Future studies should include Ca-Mg combined treatments and multi-omics analyses to further clarify their interaction mechanisms.

## 4. Materials and Methods

### 4.1. Plant Materials, Experimental Site, and Treatments

The field experiment was conducted in 2025 in Heshuye Village, Fenghua District, Ningbo City, China (29°59′ N, 121°58′ E). Fenghua is a major peach-producing area in Ningbo. The region has a subtropical monsoon climate with four distinct seasons, mild and humid conditions, annual precipitation of 1350–1600 mm, and a mean annual temperature of 16.3 °C. Soil samples were collected from the rhizosphere soil of peach trees in the experimental orchard. After air-drying and passing through a 2-mm sieve, soil physicochemical properties were determined [40]. Exchangeable Ca and Mg were extracted with ammonium acetate (NH_4_OAc) and quantified by inductively coupled plasma optical emission spectrometry (ICP-OES, ICP 6000, Thermo Fisher Scientific, Waltham, MA, USA). Analytical quality control included the use of reagent blanks, calibration curves prepared with standard solutions, and replicate measurements to ensure the accuracy and precision of the analyses. The experimental soil had the following properties: total nitrogen, 158 mg kg^−1^; available phosphorus, 305 mg kg^−1^; available potassium, 249 mg kg^−1^; organic carbon, 11.3 g kg^−1^; and organic matter, 19.4 g kg^−1^; exchangeable Mg, 33.24 mg kg^−1^; exchangeable Ca: 340.52 mg kg^−1^.

The test materials were five-year-old peach trees, and the cultivar was ‘Fenghua Yulu’, planted at a spacing of 2 m × 4 m. Calcium and magnesium treatments were applied under the same field management system to allow comparative evaluation of their effects on fruit quality. Seven foliar treatments were established: water spraying (CK), 0.1% anhydrous calcium chloride (T1), 0.2% anhydrous calcium chloride (T2), 0.3% anhydrous calcium chloride (T3), 0.1% anhydrous magnesium sulfate (T4), 0.2% anhydrous magnesium sulfate (T5), and 0.3% anhydrous magnesium sulfate (T6). The concentrations of CaCl_2_ and MgSO_4_ (0.1%, 0.2%, and 0.3%) were selected based on previous studies involving foliar mineral nutrient application in fruit crops. Different concentrations of Ca and Mg fertilizers have been reported to induce concentration-dependent responses in fruit quality and nutrient accumulation [41,42,43]. Therefore, a concentration gradient of 0.1–0.3% was designed in this study to evaluate the effects of Ca and Mg supplementation while minimizing potential foliar phytotoxicity caused by excessive nutrient concentrations. The experiment was conducted using a completely randomized design with seven foliar treatments. Each treatment included three independent replicate plots. Each plot consisted of four peach trees with similar tree age and growth vigor and was considered one experimental unit. Thus, each treatment had three independent experimental units, corresponding to three biological replicates (*n* = 3). Foliar spraying was first conducted on 5 June 2025 and repeated approximately every 10 days for a total of three applications. The first application was performed during the fruit development and enlargement stage, the second application during the stone lignification stage, and the third application during the pulp filling and rapid fruit enlargement stage. Fertilizer solutions were evenly sprayed on windless, sunny days until leaf surfaces were thoroughly wetted and water droplets began to run off. Except for the fertilizer treatments, all trees were managed according to local practices, including irrigation, pruning, and disease control. Fruits and leaves were collected on 20 July 2025. Five fruits were randomly collected from different plants in each plot, with samples collected from different orientations of the outer canopy at the middle-upper canopy position. The five fruits collected from each plot were pooled and considered as one biological replicate. The samples were washed carefully with running water to remove adhering soil and debris, packed individually, and stored at −80 °C until analysis. All chemicals and reagents used in this study were purchased from Aladdin Biochemical Technology Co., Ltd. (Shanghai, China), unless otherwise stated.

### 4.2. Determination of Leaf Mineral Elements

After the final foliar spraying, leaf samples were collected from the middle canopy. Ten to thirteen disease-free leaves were collected from each tree, and samples from four trees were pooled as one biological replicate; three biological replicates were prepared for each treatment. After thorough rinsing with deionized water, one aliquot was heated at 105 °C for 15 min, dried at 80 °C, and ground into powder for mineral nutrient analysis. A second aliquot was used immediately to determine chlorophyll content. A third aliquot was frozen in liquid nitrogen and stored at −80 °C for sucrose quantification and transcriptomic analysis. Total nitrogen in leaves was determined by the Kjeldahl method. Another 0.2 g of sample was digested with an HNO_3_-H_2_O_2_ system using a DigiBlock digestion system (EH35A, LabTech, Rotherham, UK), and the contents of phosphorus (P), potassium (K), calcium (Ca), magnesium (Mg), iron (Fe), and zinc (Zn) were determined by ICP-OES (ICP 6000, Thermo Fisher Scientific, Waltham, MA, USA).

### 4.3. Determination of Leaf Pigment Content and Sucrose Content

Chlorophyll content was determined using the 80% acetone extraction method. Fresh leaf samples (0.2 g) were cut into small pieces and immersed in 80% acetone in the dark until complete decolorization. The extract was centrifuged at 10,000 rpm for 2 min at 4 °C, and 200 µL of the supernatant was used to measure absorbance at 663 and 645 nm with a microplate reader (SPECTROstar Nano, BMG LABTECH, Ortenberg, Germany). Chlorophyll a, chlorophyll b, and total chlorophyll contents were calculated according to Arnon’s equations [44]. The sucrose content in peach leaves was determined using a plant sucrose content assay kit (BC2465, Solarbio Science & Technology Co., Ltd., Beijing, China). Briefly, fresh leaf samples were homogenized in an appropriate extraction solution, and the supernatants were collected after centrifugation for sucrose determination.

The sucrose content was quantified based on the colorimetric reaction using a microplate reader, and the absorbance was measured at the specified wavelength according to the kit protocol. The sucrose content was calculated using a sucrose standard curve and expressed as mg g^−1^ fresh weight (FW).

### 4.4. Fruit Size and Shape

The single fruit weight was determined using an electronic balance and the longitudinal and transverse diameters of fruits were measured with an electronic digital vernier caliper; the fruit shape index was calculated as the ratio of fruit longitudinal diameter to transverse diameter [45].

### 4.5. Fruit Sugar-Acid Flavor Quality

Soluble solids content (SSC) was determined using a handheld refractometer (Model: DLX-ART-CTY, Delixi Electric Co., Ltd., Yueqing, China). Soluble sugar content was determined using a modified anthrone colorimetric method [46]. Fresh peach samples (0.2 g) were homogenized with 1.5 mL ethanol under ice bath conditions. The homogenate was then incubated in a 50 °C water bath with shaking for 20 min and cooled to room temperature. After centrifugation at 12,000 rpm, 25 μL of the supernatant was transferred and sequentially mixed with 75 μL distilled water, 30 μL anthrone reagent, and 250 μL concentrated sulfuric acid. The mixture was thoroughly vortexed, heated in a water bath, and then cooled to room temperature. The absorbance was measured at 620 nm using a spectrophotometer (SPECTROstar Nano, BMG LABTECH, Ortenberg, Germany).

Titratable acidity (TA) was determined as follows. Fresh peach samples (5.0 g) were homogenized with 10 mL 0.09 mol L^−1^ NaCl standard solution. The homogenate was transferred into an Erlenmeyer flask and incubated in a water bath at 80 °C for 30 min with intermittent shaking. After cooling to room temperature, the mixture was centrifuged at 4000 rpm for 10 min. The supernatant was collected and made up to 50 mL with distilled water to obtain the TA extract, which was stored at 4 °C until analysis. For titration, 10 mL of the extract was transferred into an Erlenmeyer flask, and 50 μL of phenolphthalein indicator was added. The solution was titrated with standardized 0.1 mol L^−1^ NaOH solution until a persistent light pink color appeared that remained stable for at least 0.5 min. The volume of titrant consumed was recorded. Titratable acidity was calculated based on the consumption of NaOH and expressed as g malic acid equivalent kg^−1^ FW [47].

Sucrose, glucose, and fructose contents were determined using a modified differential anthrone colorimetric assay based on the distinct color development rates of ketoses and aldoses [46,48,49]. Determination of sugar composition content: fresh homogenized samples (1.0 g) were placed in a 50 mL centrifuge tube and extracted with 20 mL of 75% ethanol in an 80 °C water bath for 15 min. After cooling to room temperature, the mixture was centrifuged and the supernatant was transferred into a 25 mL volumetric flask. The residue was repeatedly washed with a small amount of 75% ethanol, and all extracts were combined in the same volumetric flask and made up to volume with 75% ethanol. The solution was then filtered, and the filtrate was used as the soluble sugar extract. An aliquot of 1 mL of the soluble sugar extract was transferred into a test tube and diluted with distilled water to 10 mL. Then, 2 mL of 2 mol·L^−1^ KOH was added, and the mixture was boiled in a water bath for 5 min, followed by rapid cooling to room temperature. The solution was transferred into a 25 mL volumetric flask and diluted to volume with distilled water. For sucrose determination, 1 mL of the treated extract was placed in a test tube and adjusted to 2 mL with distilled water. Then 6 mL of anthrone reagent was added, mixed thoroughly, and immediately heated in a boiling water bath for 3.5 min. The mixture was rapidly cooled in an ice bath with continuous shaking. Absorbance was measured at 640 nm (SPECTROstar Nano, BMG LABTECH, Ortenberg, Germany).

Glucose and fructose contents were determined using the anthrone colorimetric method with slight modifications. Briefly, 1 mL of soluble sugar extract was diluted to 25 mL with distilled water and used for subsequent analysis. For glucose determination, 1 mL of the diluted extract was mixed with distilled water to a final volume of 2 mL, followed by the addition of 6 mL anthrone reagent. The mixture was heated in a boiling water bath for 3.5 min, immediately cooled, and the absorbance was measured at 640 nm (E_100_). For fructose determination, the same volume of extract was reacted with 6 mL anthrone reagent at 50 °C for 3 min, followed by cooling and absorbance measurement (E_50_). The contents of glucose and fructose were calculated using the corresponding standard curves.

For the determination of calcium and magnesium contents in fruits, the peach flesh was dried, ground into powder, 0.4 g of the powder was weighed and digested with 10 mL of nitric acid, then diluted to 50 mL, and the calcium and magnesium contents were determined by inductively coupled plasma optical emission spectrometry (ICP-OES, ICP 6000, Thermo Fisher Scientific, Waltham, MA, USA).

### 4.6. Fruit Vitamin C Content and Antioxidant Properties

Total flavonoid content was determined using the aluminum chloride colorimetric method with slight modifications [50]. Determination of total flavonoid content: fresh peach fruit samples (0.2 g) were ground and extracted with 10 mL of 60% ethanol, followed by incubation in a 70 °C water bath for 1 h. After cooling, the mixture was centrifuged and the supernatant was transferred into a 25 mL volumetric flask. The residue was re-extracted with 10 mL of 60% ethanol, shaken thoroughly, and incubated again in a 70 °C water bath for 1 h. All extracts were combined, made up to volume, and filtered. An aliquot of 1.0–5.0 mL of the filtrate was transferred into a 10 mL volumetric flask, followed by the addition of 0.5 mL of 5% NaNO_2_. After shaking and standing for 6 min, 0.5 mL of 10% Al(NO_3_)_3_ solution was added. The mixture was shaken again and allowed to stand for another 6 min, then 4 mL of 4% NaOH solution was added. The volume was adjusted to 10 mL with 60% ethanol, mixed thoroughly, and allowed to stand for 15 min. The absorbance was measured at 510 nm (BioTek, Epoch2, Shoreline, WA, USA). The total flavonoid content was calculated using a rutin standard curve and mg RE g^−1^ FW.

Determination of total phenolic content: fruit samples (0.1 g) were homogenized and washed into a 25 mL volumetric flask with 20 mL of distilled water. The mixture was incubated in a boiling water bath at 100 °C for 30 min, then cooled to room temperature, made up to volume, and filtered. An aliquot of 1. 0 mL of the filtrate was transferred into a 10 mL volumetric flask, followed by the addition of 1 mL of Folin–Ciocalteu (FC) reagent and 3 mL of 7.5% sodium carbonate solution. The mixture was diluted to volume with distilled water, mixed thoroughly, and allowed to stand at room temperature for 30–60 min for color development. The absorbance was measured at 765 nm (SPECTROstar Nano, BMG LABTECH, Ortenberg, Germany) [51]. The total phenolic content was calculated using a gallic acid standard curve and expressed as mg GAE g^−1^ FW.

Vitamin C content was determined using a Vitamin C assay kit (Cat. No. TC2031, Leagen Biotechnology Co., Ltd., Beijing, China) according to the manufacturer’s instructions. The antioxidant capacity of peach flesh was evaluated by determining DPPH radical scavenging activity using a DPPH Free Radical Scavenging Capacity Assay Kit (Cat. No. M0112B, Suzhou Michy Biomedical Technology Co., Ltd., Suzhou, China).

### 4.7. Leaf Transcriptome Sequencing and Analysis

Total RNA was extracted from peach leaf samples using the RNAprep Pure Plant Kit (Tiangen, Beijing, China) according to the manufacturer’s instructions. RNA concentration and purity were determined using a NanoDrop 2000 spectrophotometer (Thermo Fisher Scientific, Wilmington, DE, USA), and RNA integrity was assessed using an Agilent 2100 Bioanalyzer system (Agilent Technologies, Santa Clara, CA, USA).

For transcriptome sequencing, three independent biological replicates were prepared for each treatment (T6 and CK), resulting in six RNA-seq libraries. A total of 1 μg of qualified RNA from each sample was used for library construction. mRNA was enriched using oligo(dT)-attached magnetic beads and subsequently fragmented. First- and second-strand cDNA were synthesized, followed by end repair, 3′ adenylation, and adapter ligation. The cDNA fragments were purified using AMPure XP beads (Beckman Coulter, Beverly, MA, USA), followed by PCR amplification. The quality of the constructed libraries was assessed before sequencing.

The libraries were sequenced on an Illumina NovaSeq 6000 platform to generate 150-bp paired-end (PE150) reads. The sequencing data were processed using the bioinformatic analysis pipeline provided by BMKCloud (Biomarker Technologies, Nanjing, China; www.biocloud.net).

Raw reads in FASTQ format were filtered to remove adapter-containing reads, reads with more than 10% ambiguous bases (N), and low-quality reads. The resulting clean reads were used for subsequent analyses. Sequencing quality was assessed based on Q20, Q30, GC content, and sequence duplication levels. A total of 41.77 Gb of clean data were obtained, with at least 6.14 Gb generated for each sample, and the Q30 percentage was ≥95.38%.

Clean reads were aligned to the peach (*Prunus persica* L. Batsch) reference genome (NCBI assembly GCF_000346465.2) using HISAT2 v2.2.1. Transcript assembly and merging were performed using StringTie v2.2.1, and the assembled transcripts were compared with the reference annotation using gffcompare v0.12.6. Alternative splicing events were analyzed using ASprofile. Based on the transcript assembly and comparison results, 1897 gene structures were optimized and 1669 novel genes were identified.

Functional annotation was performed against the NCBI non-redundant protein sequence (Nr), Swiss-Prot, Clusters of Orthologous Groups (COG/KOG), Pfam, Gene Ontology (GO), and KEGG databases. Similarity searches against protein databases were performed using DIAMOND v2.0.15. Pfam annotation was performed using HMMER/hmmscan v3.3.2, and GO annotation was performed using InterProScan v5.34-73.0.

Gene expression levels were quantified based on the mapped sequencing reads and expressed as fragments per kilobase of transcript per million fragments mapped (FPKM).

Because three biological replicates were available for each treatment, differential expression analysis between the T6 and CK groups was performed using DESeq2 v1.30.1 based on gene-level raw read counts. DESeq2 uses a negative binomial distribution model, and statistical significance was assessed using the Wald test. *p*-values were adjusted using the Benjamini–Hochberg method to control the false discovery rate (FDR). Genes with an FDR < 0.01 and an absolute fold change (|FC|) ≥ 1.5 were considered differentially expressed genes (DEGs), corresponding to |log2FC| ≥ 0.585.

GO enrichment analysis of the DEGs was performed using the clusterProfiler R package v4.4.4 based on the Wallenius non-central hypergeometric distribution. KEGG pathway enrichment analysis was performed using the BMKCloud enrichment pipeline and clusterProfiler.

### 4.8. Statistical Analysis

SPSS 18.0 (SPSS Inc., Chicago, IL, USA) was used to perform the analysis of data relating to relevant indicators, multiple comparisons were made using the ANOVA and Duncan multi-range test (*p* < 0.05). Figures were generated using OriginPro 2018 (OriginLab Corporation, Northampton, MA, USA).

## 5. Conclusions

This study showed that foliar application of Ca and Mg positively regulated leaf physiological performance and fruit quality of Fenghua Yulu peach. Ca and Mg should be interpreted as two independent foliar fertilization series rather than a combined treatment. Foliar application of CaCl_2_ and MgSO_4_ improved mineral nutrient status and leaf chlorophyll content, with the effects varying according to fertilizer type and concentration. High-concentration foliar application of calcium or magnesium enhanced fruit external and internal quality, although the strongest effects differed by element. Among the calcium treatments, 0.3% CaCl_2_ resulted in relatively higher leaf calcium accumulation and showed potential effects on improving certain fruit external quality traits within the tested concentration range. Among the magnesium treatments, 0.3% MgSO_4_ showed the most prominent effects on leaf magnesium content, soluble sugar accumulation, soluble solids content, DPPH radical scavenging activity, and overall fruit quality. Transcriptomic analysis of peach leaves suggested that magnesium foliar fertilization regulated leaf carbon metabolism by altering the expression of genes involved in starch and sucrose metabolism, including inv, sus, bglx, glgc, pgm, isa, and bam. It also upregulated genes related to phenylpropanoid, flavonoid, and lignin biosynthesis, including 4cl, ccoacomt, ccr, cad, pod, chs, and fls, thereby promoting total phenol accumulation and antioxidant capacity. Overall, within the concentration range evaluated in this study, 0.3% CaCl_2_ and 0.3% MgSO_4_ showed promising effects on nutrient status and fruit quality improvement, respectively. The potential roles of the accompanying Cl^−^ and SO_4_^2−^ ions also merit further investigation. Future studies incorporating appropriate controls could help further clarify the respective contributions of Ca^2+^, Mg^2+^, Cl^−^ and SO_4_^2−^ to the observed physiological responses.

## Figures and Tables

**Table 1:** Effects of different treatments on the external quality of fruit.

Treatment	Fruit Weight (g)	Longitudinal Diameter (mm)	Transverse Diameter (mm)	Fruit Shape Index
CK	191.3 ± 7.02 b	70.4 ± 1.93 a	69.07 ± 0.92 a	1.03 ± 0.01 a
T1	194.4 ± 6.85 ab	73 ± 0.98 a	71.14 ± 1.11 a	1.05 ± 0.02 a
T2	192.62 ± 7.99 ab	70.93 ± 2.85 a	69.52 ± 1.3 a	1.03 ± 0.01 a
T3	203.7 ± 7.69 ab	72.99 ± 2.98 a	71.72 ± 1.88 a	1.03 ± 0.01 a
T4	204.04 ± 4.71 ab	75.62 ± 4.4 a	72.23 ± 3.75 a	1.04 ± 0.02 a
T5	205.7 ± 4.24 ab	75.34 ± 1.95 a	72.61 ± 2.72 a	1.05 ± 0.02 a
T6	208.41 ± 1.46 a	75.47 ± 0.6 a	72.78 ± 1.15 a	1.03 ± 0.03 a

Data are presented as means ± SD (*n* = 3). Statistical significance was determined using one-way ANOVA followed by Duncan’s multiple range test at *p* < 0.05. Different letters indicate significant differences among treatments.

**Table 2:** Principal component loading, eigenvalues and contribution rates.

	Loading		
Index	The 1st Principal Component	The 2nd Principal Component	The 3rd Principal Component
Single fruit weight	0.38	−0.24	0.20
Longitudinal diameter	0.35	−0.37	−0.09
Transverse diameter	0.36	−0.40	0.03
Titratable acid	0.22	0.46	0.17
soluble sugar	0.38	−0.08	0.07
Glucose	0.30	0.46	0.11
Fructose	0.30	0.44	0.01
Sucrose	0.17	−0.08	0.71
Total phenols	0.30	0.05	−0.55
DPPH radical scavenging activity	0.34	0.06	−0.31
Eigenvalue	4.55	1.80	1.30
Variance contribution rate/%	45.50	17.99	12.99
Accumulated contribution rate/%	45.50	63.49	76.47

## Data Availability

The original contributions presented in this study are included in the article. Further inquiries can be directed to the corresponding authors.

## References

[1] Layne D., Bassi D. (2008). The peach: Botany, production, and uses. Hortscience.

[2] Veerappan K., Natarajan S., Chung H., Park J. (2021). Molecular Insights of Fruit Quality Traits in Peaches, *Prunus persica*. Plants.

[3] Kader A.A. (2008). Flavor quality of fruits and vegetables. J. Sci. Food Agric..

[4] Lynch J.P. (2019). Root phenotypes for improved nutrient capture: An underexploited opportunity for global agriculture. New Phytol..

[5] Seghouani M., Bravin M.N., Mollier A. (2024). Crop response to nitrogen-phosphorus colimitation: Theory, experimental evidences, mechanisms, and models. A review. Agron. Sustain. Dev..

[6] Guo J.H., Liu X.J., Zhang Y., Shen J.L., Han W.X., Zhang W.F., Christie P., Goulding K.W.T., Vitousek P.M., Zhang F.S. (2010). Significant Acidification in Major Chinese Croplands. Science.

[7] Hepler P. (2005). Calcium: A central regulator of plant growth and development. Plant Cell.

[8] de Freitas S., Shackel K., Mitcham E. (2011). Abscisic acid triggers whole-plant and fruit-specific mechanisms to increase fruit calcium uptake and prevent blossom end rot development in tomato fruit. J. Exp. Bot..

[9] Hocking B., Tyerman S.D., Burton R.A., Gilliham M. (2016). Fruit Calcium: Transport and Physiology. Front. Plant Sci..

[10] Tränkner M., Tavakol E., Jákli B. (2018). Functioning of potassium and magnesium in photosynthesis, photosynthate translocation and photoprotection. Physiol. Plant..

[11] Zhang B., Cakmak I., Feng J., Yu C., Chen X., Xie D., Wu L., Song Z., Cao J., He Y. (2020). Magnesium Deficiency Reduced the Yield and Seed Germination in Wax Gourd by Affecting the Carbohydrate Translocation. Front. Plant Sci..

[12] Zhu Z., Zhang H., Tian H., Chai G., Muhammad R., Wang Q., Liang B., Wu X. (2025). Comprehensive analysis of the effects on photosynthesis and energy balance in tomato leaves under magnesium deficiency. Plant Physiol. Biochem..

[13] Ishfaq M., Wang Y., Yan M., Wang Z., Wu L., Li C., Li X. (2022). Physiological Essence of Magnesium in Plants and Its Widespread Deficiency in the Farming System of China. Front. Plant Sci..

[14] Gong K., Wang N., Chen Y., Yu J., Kuang C., Xiong X., Wan R., Xing F., Suzuki M., Peng L. (2026). Enhancing Iron Nutrition in Citrus: Synergistic Roles of Proline-2′-deoxymugineic Acid in Root Physiology and Microbiome. J. Agric. Food Chem..

[15] Penn C.J., Camberato J.J. (2019). A Critical Review on Soil Chemical Processes that Control How Soil pH Affects Phosphorus Availability to Plants. Agriculture.

[16] Yu X., Keitel C., Zhang Y., Wangeci A.N., Dijkstra F.A. (2022). Global meta-analysis of nitrogen fertilizer use efficiency in rice, wheat and maize. Agric. Ecosyst. Environ..

[17] de Castro S.A.Q., Schjoerring J.K., Sparks D.L. (2024). Chapter Three—Foliar nitrogen and phosphorus fertilization. Advances in Agronomy.

[18] Fernández V., Eichert T. (2009). Uptake of Hydrophilic Solutes Through Plant Leaves: Current State of Knowledge and Perspectives of Foliar Fertilization. Crit. Rev. Plant Sci..

[19] Otto R., Marques J., de Carvalho H. (2021). Strategies for probing absorption and translocation of foliar-applied nutrients. J. Exp. Bot..

[20] Januszkiewicz R., Kulczycki G., Samoraj M. (2023). Foliar Fertilization of Crop Plants in Polish Agriculture. Agriculture.

[21] El-Jendoubi H., Vázquez S., Calatayud Á., Vavpetič P., Vogel-Mikuš K., Pelicon P., Abadía J., Abadía A., Morales F. (2014). The effects of foliar fertilization with iron sulfate in chlorotic leaves are limited to the treated area. A study with peach trees (*Prunus persica* L. Batsch) grown in the field and sugar beet (*Beta vulgaris* L.) grown in hydroponics. Front. Plant Sci..

[22] Erengül S., Yaman M., Tunç Y., Khadivi A. (2025). Multivariate analysis of the effect of foliar fertilisers on phytochemical and fruit traits of pomegranate (*Punica granatum* L.). J. Hortic. Sci. Biotechnol..

[23] Wu L., Wang F., Sha R., Li X., Yu K., Feng J. (2023). The Effect of N and KH_2_PO_4_ on Skin Color, Sugars, and Organic Acids of “Flame Seedless” Grape. Agronomy.

[24] Arseneault M., Cline J. (2018). AVG, NAA, boron, and magnesium influence preharvest fruit drop and fruit quality of ‘Honeycrisp’ apples. Can. J. Plant Sci..

[25] Wang G., Wang J., Han X., Chen R., Xue X. (2022). Effects of Spraying Calcium Fertilizer on Photosynthesis, Mineral Content, Sugar–Acid Metabolism and Fruit Quality of Fuji Apples. Agronomy.

[26] Marschner P. (2012). Marschner’s Mineral Nutrition of Higher Plants.

[27] Feng D., Wang X., Gao J., Zhang C., Liu H., Liu P., Sun X. (2023). Exogenous calcium: Its mechanisms and research advances involved in plant stress tolerance. Front. Plant Sci..

[28] Wang T., Tan L., Chen Z., Yang Y., Yuan Y., Zheng Z., Deng L., Zhang M., Sun G., He S. (2024). Mitigating citrus fruit cracking: The efficacy of chelated calcium or silicon foliar fertilizers in ‘Okitsu no. 58’ citrus fruit. Front. Plant Sci..

[29] Bagnazari M., Saidi M., Moharnmadi M., Khademi O., Nagaraja G. (2018). Pre-harvest CaCl_2_ and GA3 treatments improve postharvest quality of green bell peppers (*Capsicum annuum* L.) during storage period. Sci. Hortic..

[30] Val J., Fernández V. (2011). In-season calcium-spray formulations improve calcium balance and fruit quality traits of peach. J. Plant Nutr. Soil Sci..

[31] de Bang T.C., Husted S., Laursen K.H., Persson D.P., Schjoerring J.K. (2021). The molecular–physiological functions of mineral macronutrients and their consequences for deficiency symptoms in plants. New Phytol..

[32] Liu X., Hu C., Liu X., Riaz M., Liu Y., Dong Z., Tan Q., Sun X., Wu S., Tan Z. (2022). Effect of magnesium application on the fruit coloration and sugar accumulation of navel orange (*Citrus sinensis Osb.*). Sci. Hortic..

[33] Bai R., Liu H., Liu Y., Yong J.W.H. (2024). Effects of Foliar Application of Magnesium Fertilizer on Photosynthesis and Growth in Grapes. Agronomy.

[34] Nas Y., Kömürkara Zengin S. (2024). Agronomic, Biochemical, and Physiological Responses to Magnesium (Mg) Application in Tomato (*Solanum lycopersicum* L.) under Greenhouse Conditions. Horticulturae.

[35] Ali M., Hu X., Chao P., Ali S., Akram M., Naveed W., Gull S., Deng H., Mosa W., Hou Y. (2024). Magnesium’s impact on fruit quality of loquat: Insights into sugar and acid dynamics. Sci. Hortic..

[36] Su D., Yan X., Ye D., Jiang Y., Zhang J., Song B., Shi Y., Zhang W., Wu L. (2025). Magnesium fertilization promotes flavor balance and phenolic accumulation in Guanxi honey pomelo (*Citrus maxima* (Burm.) Merr.) on acidic soils. Sci. Hortic..

[37] Madani B., Mirshekari A., Yahia E. (2016). Effect of calcium chloride treatments on calcium content, anthracnose severity and antioxidant activity in papaya fruit during ambient storage. J. Sci. Food Agric..

[38] Su D., Jiang Y., Song B., Wu Z., Yan X., He Z., Ye D., Ou J., Zeng Y., Wu L. (2024). Reduced Fertilization and Magnesium Supplementation: Modulating Fruit Quality in Honey Pomelo (*Citrus maxima* (Burm.) Merr.). Plants.

[39] Tian G., Qin H., Liu C., Xing Y., Feng Z., Xu X., Liu J., Lyu M., Jiang H., Zhu Z. (2023). Magnesium improved fruit quality by regulating photosynthetic nitrogen use efficiency, carbon–nitrogen metabolism, and anthocyanin biosynthesis in ‘Red Fuji’ apple. Front. Plant Sci..

[40] Bao S.D. (2000). Soil and Agricultural Chemistry Analysis.

[41] Khan I.A., Rafiq A., Ganie S. (2019). Effect of Different Sources and Concentrations of Pre-harvest Calcium and Boron Sprays on the Quality and Yield of Apple (*Malus x domestica Borkh.*). J. Krishi Vigyan.

[42] Yang W., Zhang M., Song S., Wang L., Ren H., Liu P., Shao C., Ma F., Li M., Ma B. (2025). Effects of spraying foliar magnesium fertilizer on photosynthetic properties and fruit quality of Malus domestica Borkh. cv. ‘Fuji’. Plant Sci. J..

[43] Yogaratnam N., Johnson D.S. (1982). The Application of Foliar Sprays Containing Nitrogen, Magnesium, Zinc and Boron to Apple Trees. II. Effects on the Mineral Composition and Quality of the Fruit. J. Hortic. Sci..

[44] Arnon D.I. (1949). Copper Enzymes in Isolated Chloroplasts. Polyphenoloxidase in Beta Vulgaris. Plant Physiol..

[45] Cirilli M., Baccichet I., Chiozzotto R., Silvestri C., Rossini L., Bassi D. (2021). Genetic and phenotypic analyses reveal major quantitative loci associated to fruit size and shape traits in a non-flat peach collection (*P. persica* L. Batsch). Hortic. Res..

[46] Yemm E.W., Willis A.J. (1954). The estimation of carbohydrates in plant extracts by anthrone. Biochem. J..

[47] Ranganna S. (1986). Handbook of Analysis and Quality Control for Fruit and Vegetable Products.

[48] Jermyn M.A. (1956). A New Method for determining Ketohexoses in the Presence of Aldohexoses. Nature.

[49] Mokrasch L.C. (1954). Analysis of hexose phosphates and sugar mixtures with the anthrone reagent. J. Biol. Chem..

[50] Jia Z., Tang M., Wu J. (1999). The determination of flavonoid contents in mulberry and their scavenging effects on superoxide radicals. Food Chem..

[51] Singleton V.L., Rossi J.A. (1965). Colorimetry of Total Phenolics with Phosphomolybdic-Phosphotungstic Acid Reagents. Am. J. Enol. Vitic..

